# Perioperative factors influencing immediate and long‐term continence after robot‐assisted radical prostatectomy

**DOI:** 10.1002/bco2.70207

**Published:** 2026-04-22

**Authors:** Till Rostalski, Anja Riediger, Axel Benner, Magdalena Görtz

**Affiliations:** ^1^ Department of Urology University of Heidelberg Medical Center Heidelberg Germany; ^2^ Institute for Artificial Intelligence in Medicine University Hospital Essen Essen Germany; ^3^ Junior Clinical Cooperation Unit, Multiparametric Methods for Early Detection of Prostate Cancer German Cancer Research Center (DKFZ) Heidelberg Germany; ^4^ Faculty of Biosciences Heidelberg University Heidelberg Germany; ^5^ Division of Biostatistics German Cancer Research Center (DKFZ) Heidelberg Germany

**Keywords:** perioperative care, postoperative complications, prostatic neoplasms, robotic surgical procedures, urinary incontinence

## Abstract

**Objectives:**

This study aims to identify immediate and long‐term predictors of postoperative urinary continence recovery after robotic‐assisted radical prostatectomy (RARP) in a cohort of 1061 patients, enabling risk stratification and informing potentially modifiable perioperative strategies across patient subgroups.

**Patients and methods:**

Prospectively collected data from 1061 patients who underwent RARP between 2016 and 2021 at a single high‐volume hospital were analysed. Urinary continence was evaluated immediately after surgery (*n* = 1061), after 1 year (*n* = 797) and after 3 years (*n* = 621). Multivariable ordinal logistic regression analyses were performed for each time point.

**Results:**

Immediately after catheter removal, 34.8% of patients were continent. Younger age, shorter catheterization duration, Retzius‐sparing approach and nerve‐sparing techniques were significant predictors (*p* < 0.05). After 1 year, the continence rate was 64.2%, with shorter catheterization duration and nerve‐sparing techniques being significant. After 3 years, the continence rate was 79.1%. Only nerve‐sparing techniques remained significantly associated with continence.

**Conclusion:**

Younger age, shorter catheterization, the Retzius‐sparing approach and nerve‐sparing surgical techniques were predictive for immediate continence after RARP and represent modifiable factors that should be considered where appropriate. For long‐term continence, intraoperative nerve‐sparing is particularly crucial.

## 1 INTRODUCTION

Prostate cancer (PC) stands as the most prevalent urinary malignancy affecting men worldwide. In 2023, it accounted for approximately 29% of new cancer diagnoses in men, with the incidence expected to increase further.^1^, ^2^ Over recent years, robotic‐assisted radical prostatectomy (RARP) has emerged as one of the primary therapeutic approaches for localized PC.^3^, ^4^ While RARP is a curative‐intended treatment, it also introduces a range of complications, prominently among them being stress urinary incontinence (SUI).

Depending on the assessment criteria and timing, approximately 40%–80% of patients may experience urinary incontinence after the surgical procedure.^5^ SUI, characterized by the involuntary loss of urine during activities that exert intra‐abdominal pressure, poses a significant challenge for RARP patients. Crucially, SUI can significantly disrupt daily activities and adversely affect psychological well‐being during the postoperative period.^6^ Moreover, immediate SUI can influence the subsequent course of treatment, as the severity of incontinence at the initiation of adjuvant radiotherapy (ART) might play a pivotal role in long‐term continence recovery.^7^, ^8^ While the consequences of the delay between RARP and ART remain a subject of debate, clinicians face a dilemma of potentially postponing ART to improve continence outcomes, which could, in turn, compromise the effectiveness of radiotherapy.^9^, ^10^ Previous research has produced conflicting results concerning the precise factors that predict the onset of SUI following RARP.

A comprehensive meta‐analysis by Ficarra and colleagues in 2012 identified several predictors of post‐RARP urinary incontinence, including increasing patient age, body mass index (BMI), lower urinary tract symptoms, and prostate volume (PV).^11^ Additionally, a meta‐analysis conducted by Reeves and colleagues highlighted the impact of nerve‐sparing techniques on urinary continence outcomes.^12^ They found that avoiding damage to the nerves around the prostate significantly improves urinary continence within the first 6 months after surgery.

Multiple studies have demonstrated the beneficial effect of the Retzius‐sparing surgical approach, introduced by Galfano and colleagues in 2010, on postoperative continence.^13^, ^14^, ^15^, ^16^, ^17^ In contrast to the conventional method, this technique avoids opening the Retzius space, thereby preserving critical structures essential for urinary control and sexual function recovery. However, long‐term continence follow‐up data after RARP remain scarce.

This study sought to identify and evaluate the prognostic factors associated with both immediate and long‐term SUI following RARP on a cohort of a total of 1061 patients, aiming to provide a more comprehensive understanding of important perioperative factors for achieving continence.

## 2 PATIENTS AND METHODS

### 2.1 Patient selection

This study analysed prospectively collected data at the Department of Urology, Heidelberg University Hospital. The study included all patients who underwent RARP between 2016 and 2021 and agreed to follow‐up documentation. The total cohort consisted of 1061 patients. Continence status was available for 797 patients at the 1‐year follow‐up and for 621 patients at the 3‐year follow‐up. The study received approval from the ethical committee of the University of Heidelberg (S‐403/2012 and S‐287/2022).

### 2.2 Continence assessment

Immediate SUI was defined as the continence status directly after the removal of the transurethral catheter following RARP. Evaluation was based on a micturition/pad‐use diary and an interview at discharge. Continence/SUI was assessed postoperatively on the ward by ward physicians as part of routine postoperative care documentation. Patients were classified as continent, if they required no safety pad. The severity of SUI was graded according to standardized criteria and assessed by the responsible medical professional.^18^ SUI severity was graded using the commonly reported symptom‐based Stamey classification (after Ingelman‐Sundberg), as described in the literature (Grade I/II/III)^19^: Grade I (mild): leakage with coughing or other vigorous increases in intra‐abdominal pressure (e.g., sneezing, laughing). Grade II (moderate): leakage with less intense activity or change in position (e.g., walking, standing up, sitting up). Grade III (severe): leakage without relation to physical activity or position, that is, continuous or near‐continuous leakage. After hospital discharge, an institutional questionnaire was regularly sent by mail to monitor the progress of continence recovery. Patients underwent follow‐up care in outpatient settings, and follow‐up data were available if they responded to the questionnaires sent by Heidelberg University Hospital. Incontinence was recorded without occlusive aids, that is, based on urine loss without the use of penile clamps or other occlusive devices.

### 2.3 Evaluated parameters

For each patient, we collected the following preoperative parameters: Age, BMI, PSA level, clinical stage, prostate volume and Gleason score in the prostate tissue biopsy. Furthermore, the following perioperative/postoperative information was recorded: immediate SUI grade, surgical approach (standard, Retzius‐sparing), number of harvested lymph nodes, nerve‐sparing (none, unilateral or bilateral), bladder neck preserving, Gleason score in the prostatectomy tissue, intraoperative watertightness of anastomosis, pathological tumour stage, duration of catheterization, postoperative surgical complications, length of the hospital stay and ART (Table 1).

**TABLE 1:** Perioperative characteristics of the patient cohort.

Variable	Early continence (*n* = 1061)	1‐year continence (*n* = 797)	3‐year continence (*n* = 621)
Preoperative			
Age (years)	65.4 [60.3–70.3]	65.3 [60.3–70.1]	65.5 [60.3–70.1]
Prostate volume (mL)	39 [30–50]	38[30–50]	38 [30–50]
BMI (kg/m^2^)	27 [25–29]	27 [25–29]	27 [25–29]
PSA (ng/mL)	7.31 [5.3–11.1]	7.26 [5.4–11.2]	7.27 [5.3–11.4]
Gleason score biopsy			
6	196 (18.5%)	149 (18.8%)	114 (18.4%)
7	648 (61.1%)	478 (60.3%)	376 (60.7%)
8	116 (11.0%)	83 (10.5%)	63 (10.2%)
9	92 (8.7%)	75 (9.5%)	62 (10.0%)
10	9 (0.9%)	8 (1.0%)	4 (0.7%)
Clinical T‐stage			
T1a	3 (0.3%)	3 (0.4%)	3 (0.5%)
T1c	626 (59.0%)	476 (59.7%)	363 (58.4%)
T2	370 (34.9%)	275 (34.5%)	219 (35.3%)
T3	61 (5.8%)	42 (5.3%)	35 (5.6%)
T4	1 (0.1%)	1 (0.1%)	1 (0.2%)
Risk group (EAU)			
Low	145 (13.7%)	111 (13.9%)	87 (14.0%)
Intermediate	756 (71.3%)	566 (71.0%)	440 (70.9%)
High	160 (15.1%)	120 (15.1%)	94 (15.1%)
Intraoperative			
Surgical approach			
Retzius‐Sparing surgery	672 (63.3%)	467 (58.6%)	340 (54.8%)
Standard surgery	389 (36.7%)	330 (41.4%)	281 (45.3%)
Nerve‐sparing			
None	328 (30.9%)	238 (29.9%)	187 (30.1%)
Unilateral	160 (15.1%)	115 (14.4%)	89 (14.3%)
Bilateral	573 (54.0%)	444 (55.7%)	345 (55.7%)
Removed lymph nodes (count)	14 [8–20]	14 [8–20]	14 [8–20]
Bladder neck preservation	827 (78.0%)	623 (78.2%)	482 (77.7%)
Watertight anastomosis	977 (92.1%)	741 (93.0%)	583 (93.9%)
Postoperative			
Length of hospital stay (days)	4 [4–5]	4 [4–5]	4 [4–5]
Transurethral catheter duration (days)	11 [2–15]	12 [2–15]	13 [3–15]
Gleason score prostatectomy			
6	40 (3.8%)	27 (3.4%)	18 (2.9%)
7	898 (84.6%)	663 (83.7%)	523 (84.8%)
8	21 (2.0%)	15 (1.9%)	11 (1.8%)
9	100 (9.4%)	85 (10.7%)	63 (10.2%)
10	2 (0.2%)	2 (0.3%)	2 (0.3%)
Pathological T stage			
pT0	5 (0.5%)	3 (0.4%)	3 (0.5%)
pT2a	39 (3.7%)	26 (3.3%)	21 (3.4%)
pT2b	7 (0.7%)	5 (0.6%)	4 (0.7%)
pT2c	594 (56.0%)	438 (55.0%)	329 (53.0%)
pT3a	258 (25.3%)	215 (27.0%)	182 (29.3%)
pT3b	148 (14.0%)	110 (13.8%)	82 (13.2%)
SUI			
Continent	369 (34.8%)	512 (64.2%)	491 (79.1%)
Grade I	444 (41.9%)	237 (29.7%)	100 (16.1%)
Grade II	188 (17.7%)	37 (4.6%)	24 (3.9%)
Grade III	60 (5.7%)	11 (1.4%)	6 (1.0%)

*Note*: Statistics are reported as median [IQR] for numerical variables and total count (percentage) for categorical variables.

### 2.4 Surgical technique

The prostatectomies were conducted by nine different surgeons. Patients underwent either a standard RARP or a Retzius‐sparing RARP procedure, employing a conventional four‐arm da Vinci surgical robot. Our approach to the Retzius‐sparing RARP closely adhered to the technique initially outlined by Galfano et al.^10^.

Lymphadenectomy was performed in 842 out of 1061 patients. Nerve‐sparing was performed whenever oncological control permitted. Anastomosis was done as running suture^20^ and was tested for watertightness by intraoperative methylene blue test.^12^ A transurethral Foley catheter was placed during surgery. The duration of transurethral catheterization was left to the discretion of the surgeon. Routine radiological assessment of the anastomosis was performed by cystography before removal of the catheter.

### 2.5 Statistical analysis

To identify prognostic factors for postoperative incontinence, we performed separate multivariable ordinal logistic regression analyses using the SUI grades (I–III) as the outcome variable. The models included age, prostate volume, BMI, risk group (according to EAU), number of removed lymph nodes, length of hospital stay, transurethral catheter duration, surgical approach, pathological T stage (grouped as low [≤pT2] and high [>pT2] to improve model robustness), nerve sparing, bladder neck preservation and watertightness of the anastomosis (Table 2 for immediate continence, Table 3 for 1‐year continence and Table 4 for 3‐year continence). Radiation therapy was added in long‐term models due to its potential impact on continence recovery.

**TABLE 2:** Results of the ordinal logistic multivariable regression for immediate incontinence grade at the time of catheter removal (1061 patients).

Variable	*p*‐value	OR	CI
**Age (years)**	**<0.001**	**1.03**	**1.01–1.05**
Prostate volume (mL)	1.00	1.00	0.99–1.01
BMI (kg/m^2^)	0.67	0.99	0.96–1.03
Removed lymph nodes (count)	0.33	0.99	0.98–1.01
Length of hospital stay (days)	0.08	1.04	1.00–1.10
**Transurethral catheter duration (days)**	**<0.001**	**1.06**	**1.05–1.08**
Pathological T stage (low vs. high)	0.57	0.93	0.73–1.19
**Surgical approach (standard vs. Retzius)**	**<0.001**	**2.17**	**1.69–2.78**
Nerve sparing (unilateral vs. bilateral)	0.51	1.12	0.80–1.58
**Nerve sparing (none vs. bilateral)**	**0.001**	**1.74**	**1.24–2.43**
Bladder neck preservation (yes vs. no)	0.77	1.04	0.79–1.39
Watertight anastomosis (yes vs. no)	0.37	0.81	0.51–1.28
Risk group (EAU intermediate vs. low)	0.425	1.15	0.81–1.64
Risk group (EAU high vs. low)	0.125	1.49	0.90–2.47

*Note*: Odds ratios (OR) represent the odds of having a higher grade of incontinence for each unit increase in continuous variables or compared to the reference category for categorical variables. Pathological T stage was stratified into low (≤pT2c) and high (>pT2c) categories. Statistically significant effects (*p* < 0.05) are highlighted in bold.

**TABLE 3:** Results of the ordinal logistic multivariable regression for incontinence grade at 1‐year follow‐up (794 patients).

Variable	*p*‐value	OR	CI
Age (years)	0.47	1.01	0.99–1.03
Prostate volume (mL)	0.66	1.00	0.10–1.01
BMI (kg/m^2^)	0.95	1.00	0.96–1.04
Removed lymph nodes (count)	0.82	1.00	0.98–1.02
Length of hospital stay (days)	0.97	1.00	0.96–1.05
**Transurethral catheter duration (days)**	**0.02**	**1.02**	**1.00–1.04**
Adjuvant radiotherapy (yes vs. no)	0.26	1.23	0.86–1.78
Pathological T stage (low vs. high)	0.76	1.05	0.76–1.47
Surgical approach (standard vs. Retzius)	0.98	1.00	0.73–1.36
Nerve sparing (unilateral vs. bilateral)	0.45	1.19	0.76–1.87
**Nerve sparing (none vs. bilateral)**	**0.02**	**1.68**	**1.11–2.54**
Bladder neck preservation (yes vs. now)	0.81	1.05	0.73–1.50
Watertight anastomosis (yes vs. now)	0.76	1.09	0.62–1.90
Risk group (EAU intermediate vs. low)	0.81	1.06	0.67–1.67
Risk group (EAU high vs. low)	0.21	1.54	0.80–2.87

*Note*: Odds ratios (OR) represent the odds of having a higher grade of incontinence for each unit increase in continuous variables or compared to the reference category for categorical variables. Pathological T stage was stratified into low (≤pT2c) and high (>pT2c) categories. Statistically significant effects (*p* < 0.05) are highlighted in bold.

**TABLE 4:** Results of the ordinal logistic multivariable regression for incontinence grade at 3‐year follow‐up (616 patients).

Variable	*p*‐value	OR	CI
Age (years)	0.87	1.00	0.97–1.03
Prostate volume (mL)	0.54	1.00	0.99–1.01
BMI (kg/m^2^)	0.28	0.97	0.92–1.03
Removed lymph nodes (count)	0.37	0.99	0.97–1.01
Length of hospital stay (days)	0.88	0.99	0.92–1.08
Transurethral catheter duration (days)	0.10	1.02	1.00–1.04
Adjuvant radiotherapy (yes vs. no)	0.28	1.29	0.82–2.04
Pathological T stage (low vs. high)	0.17	0.73	0.46–1.15
Surgical approach (standard vs. Retzius)	0.21	1.31	0.86–2.00
Nerve sparing (unilateral vs. bilateral)	0.69	0.88	0.46–1.68
**Nerve sparing (none vs. bilateral)**	**0.02**	**1.91**	**1.09–3.34**
Bladder neck preservation (yes vs. no)	0.23	1.33	0.84–2.10
Watertight anastomosis (yes vs. no)	0.35	1.43	0.67–3.06
Risk group (EAU intermediate vs. low)	0.83	1.08	0.57–2.04
Risk group (EAU high vs. low)	0.79	1.13	0.47–2.70

*Note*: Odds ratios (OR) represent the odds of having a higher grade of incontinence for each unit increase in continuous variables or compared to the reference category for categorical variables. Pathological T stage was stratified into low (≤pT2c) and high (>pT2c) categories. Statistically significant effects (*p* < 0.05) are highlighted in bold.

To address potential multicollinearity, we calculated the variance inflation factor (VIF) for each variable and applied a threshold of 3. No variables exceeded this threshold, so all were retained in the final models.

Due to the ordinal nature of the SUI grade (I–III), we selected an ordinal logistic regression model for our analyses. Specifically, we chose the proportional odds model, which assumes that the effect of each predictor is constant across all categories of the outcome variable. We verified this assumption using the Brant test.

For clarity, intercept estimates from the ordinal regression models were omitted from the results tables, focusing instead on clinically interpretable odds ratios and confidence intervals for each predictor variable. The model produces odds ratios that indicate the likelihood of being in a higher incontinence grade for each unit increase in a predictor variable, with values greater than 1.0 indicating increased odds of worse incontinence.

In all analyses conducted, a *p*‐value of <0.05 was considered statistically significant. *p* values larger than 0.01 were reported to two decimal places, those between 0.01 and 0.001 to three decimal places and values smaller than 0.001 were reported as <0.001. The statistical analysis was performed using Python, with the SciPy library (version 1.11.1) and the statsmodels library (version 0.14.0).

## 3 RESULTS

### 3.1 Study cohort

Our analysis included a total of 1061 patients who were diagnosed with PC and underwent RARP between 2016 and 2021. Based on the guidelines of the European Association of Urology, 145 patients (13.7%) were classified as low‐risk, 756 patients (71.3%) as intermediate‐risk and 160 patients (15.1%) as high‐risk.^21^ The median age at the time of surgery was 65.4 years (IQR: 60.3–70.3). The median length of hospital stay was 4 days (IQR: 4–5). The median prostate volume was 39 mL (IQR: 30–50). The surgeries were carried out by nine different surgeons; however, two of them performed the large majority (82.2%).

In our cohort, 672 of 1061 patients (63.3%) underwent the Retzius‐sparing approach, while 389 patients (36.7%) received the standard RARP. Bilateral nerve‐sparing was achieved in 573 cases (54.0%), unilateral nerve‐sparing in 160 cases (15.1%) and non‐nerve‐sparing procedures in 328 cases (30.9%). Bladder‐neck preservation was accomplished in 872 cases (78.0%). The median duration of transurethral catheterization was 11 days (IQR: 2–15). Immediate postoperative continence was reported by 369 patients (34.8%), while 444 patients (41.9%) experienced grade I SUI, 188 patients (17.7%) had grade II SUI and 60 patients (5.7%) had grade III SUI.

At 1‐year follow‐up, continence status was recorded for 797 patients. Five hundred twelve (64.2%) were continent, 237 (29.7%) reported grade I SUI, 37 (4.6%) reported grade II SUI and 11 (1.4%) reported grade III SUI. At 3‐year follow‐up, data were available for 621 patients. Of these, 491 (79.1%) were continent, 100 (16.1%) reported grade I SUI, 24 (3.9%) reported grade II SUI and 6 (1.0%) reported grade III SUI (Table 1).

### 3.2 Immediate continence

To identify prognostic factors for continence following RARP, we conducted a multivariable ordinal logistic regression analysis, using clinically relevant parameters with the SUI severity as the outcome variable. The results showed significant associations between incontinence grade and patient age (OR = 1.03, *p* < 0.001), duration of postoperative transurethral catheterization (OR = 1.06, *p* < 0.001), absence of nerve‐sparing (OR = 1.74, *p* = 0.001) and the standard surgical approach (OR = 2.17, *p* < 0.001) (Table 2).

**FIGURE 1:**
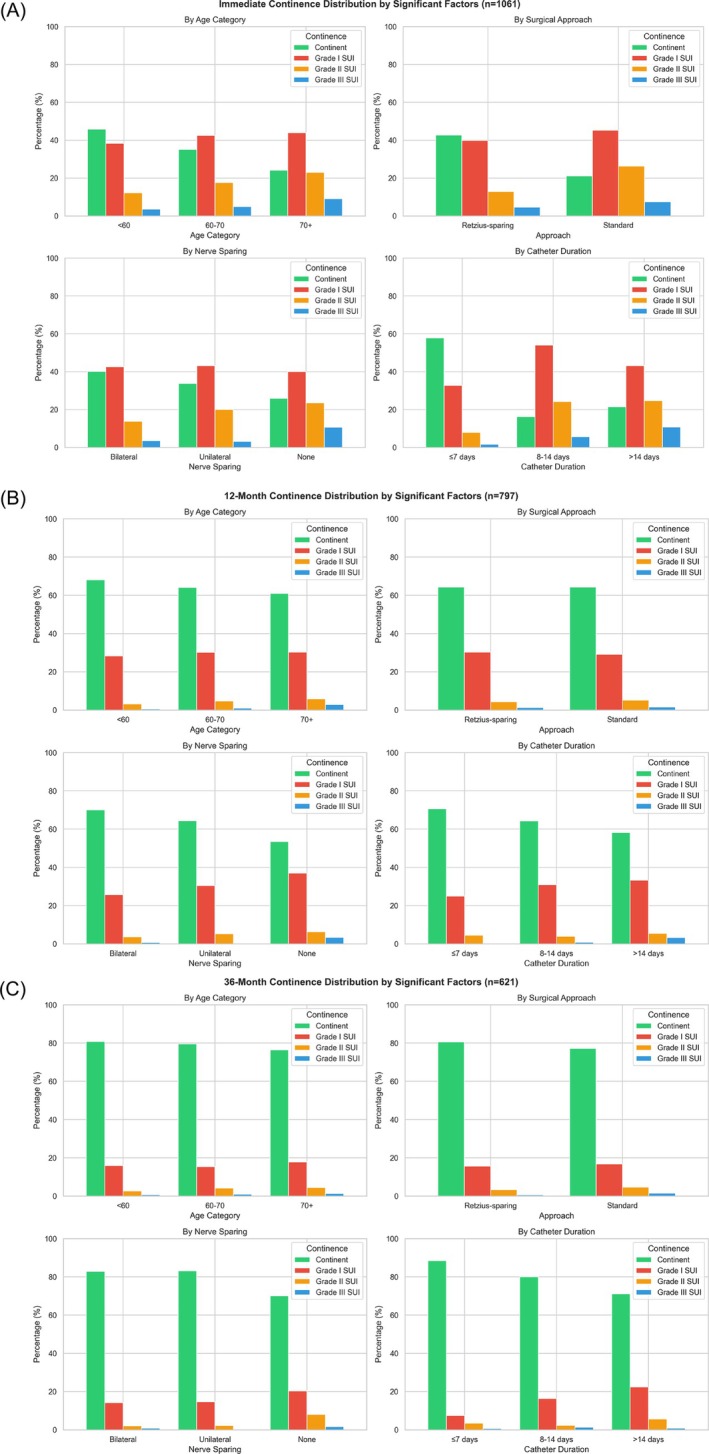
Continence outcomes stratified by initially significant clinical and surgical factors. Grouped bar charts show the distribution of continence status immediately (A), at 12 months (B) and at 36 months (C) after surgery according to age category, surgical approach, nerve‐sparing status, and postoperative catheter duration. Continence outcomes are presented as percentages and categorized as continent, Grade I SUI, Grade II SUI and Grade III SUI.

Age was positively associated with SUI severity, indicating that older patients were more likely to experience higher grades of incontinence (Figure 1). Similarly, longer transurethral catheter duration was one of the strongest predictors of SUI severity. The Retzius‐sparing technique significantly improved continence outcomes, likely by preserving critical anatomical structures. Nerve preservation appeared protective, as absence of nerve‐sparing was associated with higher SUI severity compared to bilateral nerve‐sparing. Notably, unilateral nerve‐sparing showed no significant difference from bilateral nerve‐sparing, suggesting that preserving nerves on at least one side may be beneficial for early continence.

### 3.3 Long‐term continence

To assess the predictors of long‐term continence after RARP, we analysed both 1‐ and 3‐year follow‐up data. The same variables used for immediate continence were included, with the addition of ART, due to its potential impact on continence recovery (Tables 3 and 4).

At 1‐year follow‐up, long transurethral catheterization duration (OR = 1.02, *p* = 0.02) and the absence of nerve‐sparing procedures (OR = 1.68, *p* = 0.02) were significant predictors of incontinence. Patients with prolonged catheterization were more likely to experience SUI, while those undergoing nerve‐sparing showed better continence outcomes. One hundred ninety‐four patients (24.3%) received postoperative radiation therapy within the first year, which was not significantly associated with continence outcomes (*p* = 0.26).

At 3‐year follow‐up, the impact of nerve‐sparing surgery remained significant (OR = 1.91, *p* = 0.02). The duration of transurethral catheterization approached but did not reach statistical significance (*p* = 0.1). Among patients with available follow‐up data, 186 (30.0%) received ART, which was not significantly associated with continence outcomes (*p* = 0.28).

## 4 DISCUSSION

This study investigated the influence of perioperative factors on urinary continence following RARP, revealing several insights: (1) younger patient age and the Retzius‐sparing approach were associated with improved early continence recovery, though without long‐term benefits; (2) shorter catheterization duration predicted better continence outcomes both immediately and at 1 year; and (3) nerve‐sparing was critical for both immediate and sustained continence, remaining the only significant predictor at 3 years.

In our analysis, patient age was a significant predictor of immediate incontinence, with older patients exhibiting worse outcomes. Numerous studies have described similar findings, demonstrating that younger age was associated with a quicker return to continence immediately after surgery.^22^, ^23^, ^24^, ^25^ Several studies also supported the notion that age did not correlate with overall long‐term continence outcomes.^25^, ^26^ This suggests that while younger age is beneficial for early recovery, it is also possible that continence can be restored despite advanced age, for example, through pelvic floor training.

Similarly, the Retzius‐sparing approach was significantly associated with improved immediate continence rates, consistent with previous studies by Dalela et al. and Kadhim et al., which demonstrated early continence benefits following this surgical technique.^27^, ^28^ Notably, our findings also align with the meta‐analysis by Rosenberg et al., which observed a beneficial effect of the Retzius‐sparing approach on immediate continence following transurethral catheter removal but found no significant advantage for long‐term continence.^15^ Our study adds to this body of evidence.

Interestingly, a long duration of postoperative transurethral catheterization had a strong association with immediate postoperative incontinence. Moreover, it demonstrated to be a robust predictor for incontinence at the one‐year timepoint. The catheter's main objective following RARP is to protect the anastomosis. Thus, any risks associated with too early removal of the catheter must be carefully considered, and the optimal time point of catheter removal remains a subject of debate. Only a few studies focused on the correlation of shorter catheter duration and postoperative continence. In our cohort, the median timepoint of removal was 11 days after surgery with 372 (35.1%) patients having the catheter removed at day two. In line with our results, a study by Hao and colleagues demonstrated that early catheter removal, 7 days after surgery, was associated with better continence.^29^ A study by Patel et al. showed that catheter removal at 3 or 4 days post‐RARP did not negatively affect continence but was associated with a higher incidence of acute urinary retention.^30^ In our cohort, only 11 patients (1.0%) experienced acute urinary retention, and a transurethral catheter was re‐inserted without any complications. These patients had a significantly shorter median catheter duration (2 days) compared to patients without retention (11 days) (*p* < 0.01). This supports the findings of Patel et al. and suggests that early catheter removal might be beneficial for continence but could potentially increase the risk of urinary retention.

Finally, nerve preservation emerged as a crucial predictor of both immediate and long‐term continence, if oncological safe. Our analysis showed that complete absence of nerve‐sparing was associated with worse continence outcomes across all timepoints compared to bilateral nerve‐sparing. Interestingly, unilateral nerve‐sparing provided benefits comparable to those observed with bilateral preservation. These findings align with data from Kim et al., who demonstrated that bilateral nerve‐sparing was a predictor for long‐term continence.^23^ Similarly, Srivastava et al. provided evidence for a direct correlation between the extent of nerve‐sparing and postoperative continence. Their multivariate analysis confirmed that better nerve‐sparing grades significantly predicted early continence recovery.^31^ This evidence underscores the importance of nerve preservation during RARP to optimize continence outcomes.

Our study design has several limitations. Our findings are based on data from a single centre, which may not be representative of other settings. Additionally, we were unable to obtain follow‐up data for all patients at both the 1‐ and 3‐year time points. This missing data represents an important limitation that should be considered when interpreting our results. A multicentre randomized controlled trial targeting modifiable perioperative factors to improve continence outcomes after RARP is needed to confirm our findings.

Furthermore, postoperative anti‐incontinence interventions (e.g., artificial urinary sphincter [AUS] or male sling implantation) were not systematically captured by our written institutional follow‐up questionnaire and could therefore not be fully accounted for in the analysis. Such interventions improve continence and could lead to an overestimation of spontaneous continence recovery in late follow‐up. However, large population‐based datasets suggest that only a small proportion of men undergo AUS/sling surgery after RP, with median time to first intervention around approximately 3 years. For example, a population‐based cohort from Prostate Cancer database Sweden reported that 782/26280 (3%) underwent post‐RP incontinence surgery, with a median time of 3 years after RP. Among men reporting severe patient‐reported incontinence, only about a quarter proceeded to surgery.^32^ Similarly, a population‐based cohort from Ontario reported that 2.8% underwent AUS insertion and 1.1% underwent sling placement, at a median of 2.9 years after RP.^33^

In addition, to assess the potential impact of incontinence surgery in our cohort on the continence improvement within 3 years, we performed a targeted verification focused on the 52 of 621 men who reported continence improvement from year 1 to year 3. This subgroup is the most susceptible to ‘apparent recovery’ driven by incontinence surgery, as the median time to incontinence surgery is typically around 3 years. We contacted all men still alive, successfully reached 40 of 52 men, and only 1/40 (2.5%) men reported having undergone an incontinence operation (AUS or sling). Incontinence operations thus accounted for only a small portion of observed improvement between year 1 and year 3. This finding is concordant with international registry data showing that only a few percent of RP patients undergo AUS/sling surgery overall.

However, we acknowledge that AUS/sling procedures were not systematically captured in the written questionnaire and that, while the literature suggests these interventions occur in only a minority of patients and typically around several years after RP, unmeasured interventions could still introduce a small bias in the continence estimates.

## 5 CONCLUSIONS

Nerve‐sparing surgical techniques are strongly associated with both immediate and long‐term continence after RARP, whereas younger age, shorter transurethral catheterization, and the Retzius‐sparing approach primarily influence early recovery of continence. Early catheter removal should be considered, where feasible, to optimize immediate continence recovery, and patients undergoing non‐nerve‐sparing procedures may benefit from intensified postoperative continence support.

## AUTHOR CONTRIBUTIONS

*Conceptualization*: Till Rostalski, Magdalena Görtz. *Data curation*: Till Rostalski, Magdalena Görtz. *Formal analysis*: Till Rostalski. *Investigation*: Magdalena Görtz. *Methodology*: Till Rostalski, Axel Benner. *Project administration*: Magdalena Görtz. *Resources*: Magdalena Görtz, Till Rostalski. *Software*: Till Rostalski. *Supervision*: Magdalena Görtz. *Validation*: Axel Benner. *Visualization*: Till Rostalski. *Writing – original draft*: Till Rostalski. *Writing – review and editing*: Magdalena Görtz, Anja Riediger. All authors critically reviewed, edited and added to the manuscript as well as approved the final version of the manuscript.

## CONFLICT OF INTEREST STATEMENT

No conflict declared.

## ETHICS STATEMENT

The study received approval from the ethical committee of the University of Heidelberg (S‐403/2012 and S‐287/2022).

## DECLARATION OF GENERATIVE AI AND AI‐ASSISTED TECHNOLOGIES IN THE WRITING PROCESS

During the preparation of this work, the authors used ChatGPT‐4o in order to improve language and readability. After using this tool, the authors reviewed and edited the content as needed and take full responsibility for the content of the publication.

## Data Availability

The datasets generated and analysed during the current study are not publicly available due to the sensitive nature of patient information but are available from the corresponding author upon reasonable request and subject to ethical and legal restrictions.

## References

[1] James ND , Tannock I , N'Dow J , Feng F , Gillessen S , Ali SA , et al. The Lancet commission on prostate cancer: planning for the surge in cases. Lancet. 2024;403(10437):1683–1722. 10.1016/S0140-6736(24)00651-2 38583453 PMC7617369

[2] Siegel RL , Miller KD , Wagle NS , Jemal A . Cancer statistics, 2023. CA Cancer J Clin. 2023;73(1):17–48. 10.3322/caac.21763 36633525

[3] Cooperberg MR , Broering JM , Carroll PR . Time trends and local variation in primary treatment of localized prostate cancer. J Clin Oncol. 2010;28(7):1117–1123. 10.1200/JCO.2009.26.0133 20124165 PMC2834465

[4] Herlemann A , Cowan JE , Washington SL 3rd , Wong AC , Broering JM , Carroll PR , et al. Long‐term prostate cancer‐specific mortality after prostatectomy, brachytherapy, external beam radiation therapy, hormonal therapy, or monitoring for localized prostate cancer. Eur Urol. 2024;85(6):565–573. 10.1016/j.eururo.2023.09.024 37858454

[5] Singla N , Singla AK . Post‐prostatectomy incontinence: etiology, evaluation, and management. Turk J Urol. 2014;40(1):1–8. 10.5152/tud.2014.222014 26328137 PMC4548645

[6] Kirschner‐Hermanns R , Jakse G . Quality of life following radical prostatectomy. Crit Rev Oncol Hematol. 2002;43(2):141–151. 10.1016/S1040-8428(02)00026-4 12191736

[7] Bresolin A , Garibaldi E , Faiella A , Cante D , Vavassori V , Waskiewicz JM , et al. Predictors of 2‐year incidence of patient‐reported urinary incontinence after post‐prostatectomy radiotherapy: evidence of dose and fractionation effects. Front Oncol. 2020;10:1207. 10.3389/fonc.2020.01207 32850354 PMC7396712

[8] Munoz F , Sanguineti G , Bresolin A , Cante D , Vavassori V , Waskiewicz JM , et al. Predictors of patient‐reported incontinence at adjuvant/salvage radiotherapy after prostatectomy: impact of time between surgery and radiotherapy. Cancers (Basel). 2021;13(13):3243. 10.3390/cancers13133243 34209562 PMC8269132

[9] Kowalczyk KJ , Gu X , Nguyen PL , Lipsitz SR , Trinh QD , Lynch JH , et al. Optimal timing of early versus delayed adjuvant radiotherapy following radical prostatectomy for locally advanced prostate cancer. Urol Oncol. 2014;32(3):303–308. 10.1016/j.urolonc.2013.09.004 24321259

[10] Parker CC , Clarke NW , Cook AD , Kynaston HG , Petersen PM , Catton C , et al. Timing of radiotherapy after radical prostatectomy (RADICALS‐RT): a randomised, controlled phase 3 trial. Lancet. 2020;396(10260):1413–1421. 10.1016/S0140-6736(20)31553-1 33002429 PMC7616947

[11] Ficarra V , Novara G , Rosen RC , Artibani W , Carroll PR , Costello A , et al. Systematic review and meta‐analysis of studies reporting urinary continence recovery after robot‐assisted radical prostatectomy. Eur Urol. 2012;62(3):405–417. 10.1016/j.eururo.2012.05.045 22749852

[12] Reeves F , Preece P , Kapoor J , Everaerts W , Murphy DG , Corcoran NM , et al. Preservation of the neurovascular bundles is associated with improved time to continence after radical prostatectomy but not long‐term continence rates: results of a systematic review and meta‐analysis. Eur Urol. 2015;68(4):692–704. 10.1016/j.eururo.2014.10.020 25454614

[13] Galfano A , Ascione A , Grimaldi S , Petralia G , Strada E , Bocciardi AM . A new anatomic approach for robot‐assisted laparoscopic prostatectomy: a feasibility study for completely intrafascial surgery. Eur Urol. 2010;58(3):457–461. 10.1016/j.eururo.2010.06.008 20566236

[14] Phukan C , McLean A , Nambiar A , et al. Retzius sparing robotic assisted radical prostatectomy vs. conventional robotic assisted radical prostatectomy: a systematic review and meta‐analysis. World J Urol. 2020;38(5):1123–1134. 10.1007/s00345-019-02798-4 31089802

[15] Rosenberg JE , Jung JH , Edgerton Z , et al. Retzius‐sparing versus standard robotic‐assisted laparoscopic prostatectomy for the treatment of clinically localized prostate cancer. Cochrane Database Syst Rev. 2020;2020(8):CD013641. 10.1002/14651858.CD013641.pub2 PMC7437391 32813279

[16] Qiu X , Li Y , Chen M , Xu L , Guo S , Marra G , et al. Retzius‐sparing robot‐assisted radical prostatectomy improves early recovery of urinary continence: a randomized, controlled, single‐blind trial with a 1‐year follow‐up. BJU Int. 2020;126(5):633–640. 10.1111/bju.15195 32741099

[17] Umari P , Eden C , Cahill D , Rizzo M , Eden D , Sooriakumaran P . Retzius‐sparing versus standard robot‐assisted radical prostatectomy: a comparative prospective study of nearly 500 patients. J Urol. 2021;205(3):780–790. 10.1097/JU.0000000000001435 33086025

[18] Abrams P , Cardozo L , Fall M , Griffiths D , Rosier P , Ulmsten U , et al. The standardisation of terminology in lower urinary tract function: report from the standardisation sub‐committee of the International Continence Society. Urology. 2003;61(1):37–49. 10.1016/S0090-4295(02)02243-4 12559262

[19] Mikos T , Roussos N , Theodoulidis I , Anthoulakis C , Grimbizis GF . Does pre‐operative grade of stress urinary incontinence severity affect the post‐operative outcome? A systematic review. Int Urogynecol J. 2025;36(10):1935–1949. 10.1007/s00192-025-06275-y 40990980 PMC12618355

[20] Van Velthoven RF , Ahlering TE , Peltier A , Skarecky DW , Clayman RV . Technique for laparoscopic running urethrovesical anastomosis: the single knot method. Urology. 2003;61(4):699–702. 10.1016/S0090-4295(02)02543-8 12670546

[21] Cornford P , van den Bergh RCN , Briers E , den Van Broeck T , Brunckhorst O , Darraugh J , et al. EAU‐EANM‐ESTRO‐ESUR‐ISUP‐SIOG guidelines on prostate cancer‐2024 update. Part I: screening, diagnosis, and local treatment with curative intent. Eur Urol. 2024;86(2):148–163. 10.1016/j.eururo.2024.03.027 38614820

[22] Lee DJ , Cheetham P , Badani KK . Predictors of early urinary continence after robotic prostatectomy. Can J Urol. 2010;17(3):5200–5205. 20566014

[23] Kim JJ , Ha YS , Kim JH , Jeon SS , Lee DH , Kim WJ , et al. Independent predictors of recovery of continence 3 months after robot‐assisted laparoscopic radical prostatectomy. J Endourol. 2012;26(10):1290–1295. 10.1089/end.2012.0117 22651546 PMC3466068

[24] Singh V , Sharma K , Choudhary GR , Singh M , Tripathi SS , Bhirud DP , et al. Correlation of urinary continence recovery with various factors after robot assisted radical prostatectomy. Urologia. 2024;91(1):141–146. 10.1177/03915603231191269 37632409

[25] Zorn KC , Mendiola FP , Rapp DE , Mikhail AA , Lin S , Orvieto MA , et al. Age‐stratified outcomes after robotic‐assisted laparoscopic radical prostatectomy. J Robot Surg. 2007;1(2):125–132. 10.1007/s11701-007-0009-y 25484948 PMC4247449

[26] Leyh‐Bannurah SR , Wagner C , Schuette A , Liakos N , Karagiotis T , Mendrek M , et al. Feasibility of robot‐assisted radical prostatectomy in men at senior age >/=75 years: perioperative, functional, and oncological outcomes of a high‐volume center. Aging Male. 2022;25(1):8–16. 10.1080/13685538.2021.2018417 34957914

[27] Dalela D , Jeong W , Prasad MA , Sood A , Abdollah F , Diaz M , et al. A pragmatic randomized controlled trial examining the impact of the Retzius‐sparing approach on early urinary continence recovery after robot‐assisted radical prostatectomy. Eur Urol. 2017;72(5):677–685. 10.1016/j.eururo.2017.04.029 28483330

[28] Kadhim H , Ang KM , Tan WS , Nathan A , Pavan N , Mazzon G , et al. Retzius‐sparing technique independently predicts early recovery of urinary continence after robot‐assisted radical prostatectomy. J Robot Surg. 2022;16(6):1419–1426. 10.1007/s11701-022-01383-z 35192106

[29] Hao H , Chen X , Liu Y , Si L , Chen Y , Zhang M , et al. The impact of catheter removal time on urinary continence and overactive bladder symptoms after robot‐assisted radical prostatectomy: a retrospective analysis of consecutive 432 cases from a single institution. Transl Androl Urol. 2022;11(10):1389–1398. 10.21037/tau-22-397 36386260 PMC9641063

[30] Patel R , Lepor H . Removal of urinary catheter on postoperative day 3 or 4 after radical retropubic prostatectomy. Urology. 2003;61(1):156–160. 10.1016/S0090-4295(02)02105-2 12559288

[31] Srivastava A , Chopra S , Pham A , Sooriakumaran P , Durand M , Chughtai B , et al. Effect of a risk‐stratified grade of nerve‐sparing technique on early return of continence after robot‐assisted laparoscopic radical prostatectomy. Eur Urol. 2013;63(3):438–444. 10.1016/j.eururo.2012.07.009 22901982

[32] Ventimiglia E , Folkvaljon Y , Carlsson S , Bratt O , Montorsi F , Volz D , et al. Nationwide, population‐based study of post radical prostatectomy urinary incontinence correction surgery. J Surg Oncol. 2018;117(2):321–327. 10.1002/jso.24816 28876467 PMC5873254

[33] Nam RK , Herschorn S , Loblaw DA , Liu Y , Klotz LH , Carr LK , et al. Population based study of long‐term rates of surgery for urinary incontinence after radical prostatectomy for prostate cancer. J Urol. 2012;188(2):502–506. 10.1016/j.juro.2012.04.005 22704098

